# Theabrownin triggers DNA damage to suppress human osteosarcoma U2OS cells by activating p53 signalling pathway

**DOI:** 10.1111/jcmm.13742

**Published:** 2018-07-11

**Authors:** Wangdong Jin, Li Zhou, Bo Yan, Li Yan, Fucun Liu, Peijian Tong, Wenhua Yu, Xiaoqiao Dong, Li Xie, Jin Zhang, Yiqiao Xu, Chunqi Li, Qiang Yuan, Letian Shan, Thomas Efferth

**Affiliations:** ^1^ The First Affiliated Hospital Zhejiang Chinese Medical University Hangzhou China; ^2^ Department of Orthopaedics Changzheng Hospital, Second Military Medical University Shanghai China; ^3^ Hangzhou First People's Hospital Hangzhou China; ^4^ Analysis Center of Agrobiology and Environmental Science Zhejiang University Hangzhou China; ^5^ Theabio Co., Ltd Hangzhou China; ^6^ Hunter Biotechnology, Inc Hangzhou China; ^7^ Department of Pharmaceutical Biology Institute of Pharmacy and Biochemistry Johannes Gutenberg University Mainz Germany

**Keywords:** DNA damage, osteosarcoma, P53, theabrownin, zebrafish

## Abstract

Osteosarcoma becomes the second leading cause of cancer death in the younger population. Current outcomes of chemotherapy on osteosarcoma were unsatisfactory to date, demanding development of effective therapies. Tea is a commonly used beverage beneficial to human health. As a major component of tea, theabrownin has been reported to possess anti‐cancer activity. To evaluate its anti‐osteosarcoma effect, we established a xenograft model of zebrafish and employed U2OS cells for in vivo and in vitro assays. The animal data showed that TB significantly inhibited the tumour growth with stronger effect than that of chemotherapy. The cellular data confirmed that TB‐triggered DNA damage and induced apoptosis of U2OS cells by regulation of Mki67, PARP, caspase 3 and H2AX, and Western blot assay showed an activation of p53 signalling pathway. When *P53* was knocked down by siRNA, the subsequent downstream signalling was blocked, indicating a p53‐dependent mechanism of TB on U2OS cells (p53 wt). Using osteosarcoma cell lines with p53 mutations (HOS, SAOS‐2 and MG63), we found that TB exerted stronger inhibitory effect on U2OS cells than that on p53‐mut cell lines, but it also exerted obvious effect on SAOS‐2 cells (p53 null), suggesting an activation of p53‐independent pathway in the p53‐null cells. Interestingly, theabrownin was found to have no toxicity on normal tissue *in vivo* and could even increase the viability of p53‐wt normal cells. In sum, theabrownin could trigger DNA damage and induce apoptosis on U2OS cells via a p53‐dependent mechanism, being a promising candidate for osteosarcoma therapy.

## 1 INTRODUCTION

Osteosarcoma (OS), a very aggressive intra‐osseous neoplasm, is one of the most common primary malignant bone diseases that severely threatens the health of children, adolescents and young adults.^1^ It exhibits a predilection to occur in the metaphysis of long bones, and commonly occurs in the distal femur (30%‐43%), proximal tibia (15%‐23%) or proximal humerus (10%‐15%), which represent sites containing the most proliferative growth plates.^2^, ^3^ OS is derived from primitive mesenchymal stem cells mutated in the process of differentiation towards osteoblasts, with highly invasive and distant metastatic potential.^4^ It is prone to hematogenous metastasis at early onset and after surgery. Approximately 15% to 20% of patients have clinically detectable metastases with more than 85% of occurrence in the lungs.^2^, ^5^ The world‐wide annual incidence of OS is 3.1 per million for all ages and 4.4 per million for individuals <25 years of age.^6^ Approximately 400 new cases of OS are annually diagnosed in the US and 0.2~3/100 000 patients per year (0.8‐11/100 000 patients per year in the age of 15‐19 years) are reported as overall OS incidence in the EU.^7^, ^8^ It becomes the third most common malignancy and the second leading cause of cancer‐related deaths in both children and young adults.^8^, ^9^

The SEER (Surveillance, Epidemiology and End Results) database revealed that long‐term clinical outcomes for children and young adults with OS have changed very little over the past 30 years.^10^ At present, surgery with chemotherapy is the first‐line treatment.^11^ Surgical resection of the primary tumour with adequate margins is an essential component of the curative strategy for OS patients.^12^ Nevertheless, surgical results in large bone defects of the affected limb and complex skeletal rebuilding limits its application.^13^ The gold standard of chemotherapy regimens includes high doses of cis‐platinum, doxorubicin, etoposide, ifosfamide, methotrexate and combinations of these drugs.^14^, ^15^ Unfortunately, short‐ and long‐term collateral side effects of the chemotherapy leave both clinicians and patients unsatisfied.^16^ Acute toxicities such as cardiac dysfunction, ototoxicity, infertility, myelosuppression, mucositis and secondary malignancies are frequently associated with high doses of chemotherapy, and early or late cardiac failure or sepsis following febrile neutropenia has been the major reasons for deaths caused by chemotherapeutics.^17^, ^18^ Moreover, OS may be inherently resistant to chemotherapy or become unresponsive to these drugs, which occurs in 35%‐45% of patients.^16^, ^19^ Thus, the outcome of chemotherapy remains disappointing with overall 5‐year survival rate of only about 20%,^20^, ^21^, ^22^ and effective therapies with little side effects are urgently required.

As one of the most traditional and commonly consumed non‐alcoholic beverage in the world next to water, tea (fresh leaves of *Camellia sinensis* (L) O. Kuntze) is used not only for health promotion but also medicinal purposes. It has been reported to possess antioxidant, anti‐inflammatory, anti‐proliferative and anti‐angiogenesis activities which are potentially significant to the prevention and treatment of various forms of diseases, such as cancer.^23^, ^24^ Oral intake of tea reduces the risk of many cancer incidences, including breast cancer, liver cancer, oral cancer, etc., with little adverse events.^25^, ^26^, ^27^, ^28^ Apart from the chemopreventive effect, tea also exerts chemotherapeutic effects on cancer by inducing apoptosis.^24^, ^28^, ^29^, ^30^ Theabrownin (TB), theaflavin (TF) and thearubigin (TR) are the main components of tea, which determine tea′s colour, taste and bioactivity.^31^ TB is a reddish‐brown material with the highest water solubility. It has significant cholesterol‐lowering activity, relieves fatigue and reduces blood lipid levels.^32^ TB comprises of a family of macromolecules transformed from polyphenols, and is considered superior to TF or TR in physicochemical and medicinal properties. Previously, we reported that TB possessed strong pro‐apoptotic and cell cycle arresting effects on human carcinoma cells, making it a promising candidate for cancer therapy.^33^, ^34^ To determine its anti‐OS effect, this study employed U2OS cells and performed in vivo and in vitro assays.

## 2 MATERIALS AND METHODS

### 2.1 Chemicals and reagents

Theabrownin (>90% of purity) was purchased from Theabio Co., Ltd (Hangzhou, China) (Batch number: 20151105001). Roswell Park Memorial Institute (RPMI) 1640 medium, MEM medium, fetal bovine serum (FBS), and 0.25% trypsin were purchased from Gibco BRL (Grand Island, NY, USA). 3‐(4,5‐dimethylthiazol‐2‐yl)‐2,5‐diphenyltetrazolium bromide (MTT), dimethyl sulfoxide (DMSO), McCOY′s 5A medium,and acridine orange (AO) were purchased from Sigma (St. Louis, MO, USA). STEMPRO^®^ hMSC serum free medium was purchased from ThermoFisher Scientific (Rockford, IL, USA). CM‐Dil dye was obtained from Molecular Probes (Leiden, the Netherlands). Annexin‐V:FITC apoptosis detection kit was purchased from BD Biosciences (NJ, USA). In Situ Cell Death Detection Kit was purchased from Roche Molecular Biochemicals (Mannheim, Germany). ProLong^®^ Diamond Antifade Mountant with DAPI was purchased from Invitrogen (CA, USA). All antibodies were from Cell Signaling Technology (CST, MA, USA).

### 2.2 Cell line and culture

The human OS U2OS, SAOS‐2, HOS, MG63 cell lines and 1 marrow mesenchymal stem cells (BMSC) were obtained from Shanghai Cell Bank of Chinese Academy of Sciences (Shanghai, China). U2OS, SAOS‐2, HOS and MG63 cells were cultured, respectively, in RPMI‐1640, McCOY′s 5A and MEM (HOS and MG63) medium containing 10%‐5% FBS at 37°C in a humidified 5% CO_2_ incubator. BMSC was cultured in STEMPRO^®^ hMSC serum free medium at the above condition. All mediums were daily changed and the cells were treated with TB in their logarithmic growth phase.

### 2.3 Zebrafish

The zebrafish wide‐type AB strain was purchased from the China Zebrafish Resource Center (CZRC), Institute of Hydrobiology, CAS (Wuhan, China) and bred by Hunter Biotechnology, Inc. (Hangzhou, China). The fishes were accredited by the Association for Assessment and Accreditation of Laboratory Animal Care (AAA LAC) International (SYXK2012‐0171). After natural pair‐mating and reproduction, larval zebrafish (2 dpf, days post fertilization) were generated and housed in a light‐controlled aquaculture facility with a standard 14:10 hours day/night photoperiod and fed with live brine shrimp twice a day and dry flakes once a day. The temperature of fish water was maintained at 28°C (0.2% instant ocean salt, pH6.9‐7.2, conductivity 480‐510 μS/cm and hardness 53.7‐71.6 mg/L CaCO_3_).

### 2.4 TB dose range determination in zebrafish

Totally 180 larval zebrafish (3 dpf) were randomly divided into 6 groups (30 fishes each) and cultured into 6‐well plates (Nest Biotech, China) in 3 mL fresh fish water. TB powders were dissolved into the plates at 0, 200, 400, 1000, 1500 and 2000 μg/m, respectively, for 24 hours. Thereafter, fishes were subjected to visual observation under a stereoscopic microscope to record the mortality. The mortality curve was generated using Origin 8.0 (OriginLab, Northampton, MA, USA), and the MNLC and NOAEL of TB was determined. According to the preliminary studies, the doses at 1/10 NOAEL, 1/3 NOAEL and NOAEL were used as low, middle and high dose of TB, respectively, for subsequent experiments.

### 2.5 Zebrafish U2OS xenograft model establishment and TB treatment

U2OS cells were stained with CM‐Dil (red fluorescence) at a 1:1000 dilution according to the manufacturer's protocol and microinjected into the yolk sac of larval zebrafish (2 dpf) at a dose of 200 cells/fish. After tumour growth for another 24 hours, all treated zebrafish were visually observed under a fluorescence microscope (AZ100, Nikon, Tokyo, Japan) for model verification.

### 2.6 Anti‐OS effect evaluation of TB

U2OS‐xenografted larval zebrafish (3 dpf) were grouped into 5 groups (30 fishes per group) and distributed into 6‐well plates in 3 mL fresh fish water. TB powders were dissolved into the wells at doses of 1/10 NOAEL, 1/3 NOAEL and NOAEL, respectively, as treatment groups. The untreated wells were used as model group, and the wells treated with cis‐platinum at its NOAEL level (75 μmol/L) were used as positive control group. After 24 hours treatment (3 dpf to 4 dpf), 10 zebrafish of each group were randomly selected for visual observation and imaging acquisition under fluorescence microscope using Nikon NIS‐Elements D 3.10 system. The fluorescence intensity (S) of U2OS‐developed tumour mass in each zebrafish was detected and the inhibitory rate was calculated as: Inhibitory rate (%) = [1−(TB treated S/untreated S)] × 100%.

Totally, 150 normal larval zebrafish were randomly divided into 5 groups (30 fishes each) and cultured as above. TB powders were dissolved into the wells at doses of 1/10 NOAEL, 1/3 NOAEL, NOAEL and MNLC, respectively. The untreated group and the cis‐platinum (75 μmol/L) treated group were used as normal control and positive control, respectively. After 24 hours treatment, all zebrafish were cleaned and stained with AO (acridine orange, green) to observe cell apoptosis under fluorescence microscope. The fluorescence intensity (S) of apoptotic cells in each zebrafish was detected and the pro‐apoptosis rate was calculated as: pro‐apoptosis rate (%) = [treated S/untreated S−1] × 100%.

### 2.7 Cell viability assay

Viabilities of TB‐treated cells (U2OS, SAOS‐2, HOS, MG63, BMSC) was determined by MTT assay as described in our previous report.^34^ Cells were seeded on 96‐well plates with density of 1 × 10^4^ cells/well in 200 μL medium for 24 hours and then treated with TB at different concentrations (0, 10, 20, 30, 40, 50, 60, 80 μg/mL) for 24 and 48 hours. Each 20 μL MTT solution (5.0 mg/mL) was added in each well and incubated at 37°C for 4 hours. Then 150 μL DMSO was added in each well to dissolve the MTT formazan crystals and the optical density (OD) value was measured at 490 nm using Synergy H1 microplate reader (BioTek, Winooski, VT, USA). Inhibitory rate (%) = [1−(TB‐treated OD/untreated OD)] × 100%. The 50% inhibitory concentrations (IC_50_) for 24 and 48 hours treatment were calculated by regression analysis. The 1/4 IC_50_, 1/2 IC_50_ and IC_50_ were selected as the low, medium and high concentrations of TB for following tests.

### 2.8 Cell morphology and DAPI staining

The TB‐treated U2OS cells at 24 hours were washed with phosphate‐buffered saline (PBS) thrice and fixed with 4% paraformaldehyde in PBS for 30 minutes at room temperature. Then the cells were permeabilized with 0.5% Triton X‐100 in PBS for 10 minutes. An aliquot of the cells were mounted using ProLong^®^ Diamond Antifade Mountant with DAPI in dark. The unstained and stained cells were observed under a fluorescence microscope (Carl Zeiss, Göttingen, Germany). Five coverslips were used as replicates of each group and the apoptotic nuclei of cells were visualized.

### 2.9 Flow cytometry

TB‐induced apoptosis of U2OS cells was determined by flow cytometry using an Annexin‐V/PI method, according to the manufacturer's protocol. Briefly, U2OS cells were seeded on 6‐well plates with density of 2.5 × 10^5^ cells/well for 24 hours and then were treated with TB at low, medium and high concentrations for another 24 hours. Thereafter, the cells were harvested and washed twice with cold PBS, and then labelled with FITC Annexin V and PI in binding buffer. Fluorescence intensity of the cells was detected by BD Accuri^TM^ C6 (BD, Franklin Lakes, NJ, USA). The analysis was replicated thrice and the apoptosis rate (%) for each TB treatment was calculated.

### 2.10 TUNEL and immunofluorescence assay

Apoptotic cells were observed by in situ terminal deoxynucleotidyl transferase dUTP nick end labelling (TUNEL) assay using In Situ Cell Death Detection Kit, Fluorescein (Roche, Mannheim, Germany). Briefly, U2OS cells (4 × 10^4^) were fixed with 4% paraformaldehyde for 1 hours at room temperature and permeabilized with 0.1% sodium citrate containing 0.1% Triton X‐100 at 4°C for 2 minutes. TUNEL reaction mixture was added on the cells and incubated at 37°C for 60 minutes in the dark. Then the samples were observed under fluorescence microscope using an excitation wavelength of 450‐500 nm and detection wavelength of 515‐565 nm for TUNEL assay. For immunofluorescence assay, the fixed and permeabilized U2OS cells were blocked with 1% BSA in Tris‐buffered saline‐Tween 20 (TBST) solution at 4°C for 1 hours, and then incubated with antibodies at 4°C overnight. The cells were incubated with secondary antibody at 4°C for 1 hours in the dark and observed under the fluorescence microscope. The cells incubated with antibody of p‐H2AX were observed under a Leica TCS SP5 confocal laser scanning microscope (Leica, Heidelberg, Germany) at settled excited wavelength (DAIP 405 nm, FITC 488 nm) and consistent detecting wave band (DAPI 420‐470 nm, FITC 503‐550 nm). After semi‐automatic segmentation based on the distribution of fluorescent density, image processing including number counting and intensity measuring were performed by IMARIS software package (Bitplane, Zurich, Switzerland). At least, 9 repeats were carried out for each treatment in the measurement of fluorescence density.

### 2.11 Western blot analysis

After TB treatment, total proteins of the U2OS cells (1.5 × 10^6^) were extracted using a lysis buffer (50 mmol/L Tris‐HCl, pH 7.4, 150 mmol/L NaCl, 1 mmol/L EDTA, 1% Triton, 0.1% SDS) with proteinase inhibitor cocktail (Bimake, Houston, USA) for 30 minutes on ice. The proteins were separated by a denaturing sodium dodecyl sulphate polyacrylamide gel electrophoresis (SDS‐PAGE, 6%‐12%) and then transferred onto a nitrocellulose membrane (Sartorius Stedim, Goettingen, Germany). The membrane was blocked with 5% non‐fat milk for 2 hours, followed by overnight incubation at 4°C with the primary antibodies. Following incubation with peroxidase‐conjugated goat anti‐rabbit/mouse IgG at room temperature for 2 hours, proteins were visualized using Western Lightning^®^ Plus ECL (Perkin Elmer, Waltham, Massachusetts, USA) and detected using X‐film (Kodak, Tokyo, Japan) and scanned.

### 2.12 Small interfering RNA (siRNA) transfection treatment

For transient knockdown of the *TP53* gene, 2 *TP53* siRNAs and non‐targeting control siRNA (GenePharma, Shanghai, China) were transfected into U2OS cells using Lipofectamine RNAiMAX Transfection Reagent (Thermo, Waltham, Massachusetts, USA) according to the manufacturer's instruction. The siRNA targeting sequences of *P53* are: 5′‐GACUCCAGUGGUAAUCUAC‐3′ and 5′‐GUAGAUUACCACUGGAGUC‐3′.

### 2.13 Statistical analysis

Data were expressed as mean ± SD and subjected to one‐way ANOVA, followed by Fisher's least significant difference (LSD) comparison. All analyses were performed using an updated version of DPS software.

## 3 RESULTS

### 3.1 MNLC (maximal‐no‐lethal‐concentration) and NOAEL (no‐observed‐adverse‐effect‐level) of TB

**Figure 1:**
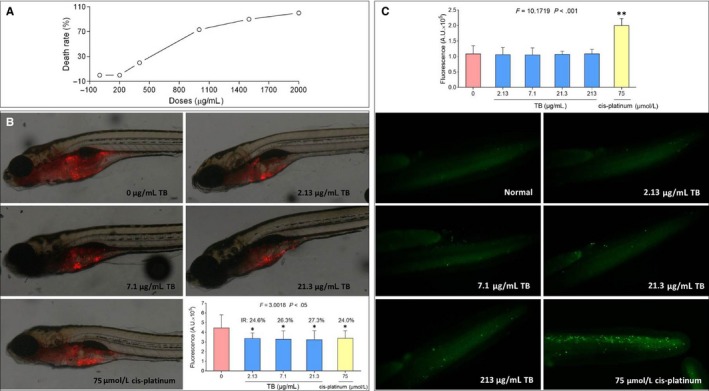
Effect of TB on U2OS‐xenotransplanted larval zebrafish and normal zebrafish. A, TB‐induced mortality curve between 3 and 4 dpf (n* *=* *30). B, Visual observation of U2OS‐xenotransplanted zebrafish (4 dpf) upon treatment with TB or cis‐platinum, and the fluorescent area (red) represents the osteosarcoma tumour. C, Visual observation of normal zebrafish upon treatment with TB or cis‐platinum by AO staining, and the fluorescent spots (green) are apoptotic cells

The mortality curve of larval zebrafish is shown in Figure 1A (upper). TB‐induced deaths were observed at dose of 400 μg/mL, and no fish survived with TB at a dose of 2000 μg/mL, indicating a dose‐dependent mortality. The MNLC of TB was thereby estimated as 213 μg/mL, using sigmoidal regression (Origin 8.0 software). During the double‐blind observation, NOAEL of TB was calculated as 21.3 μg/mL (1/10 MNLC). According to the preliminary study, 1/10 NOAEL (2.13 μg/mL), 1/3 NOAEL (7.1 μg/mL) and NOAEL (21.3 μg/mL) were adopted as low, middle and high doses for the subsequent experiments.

### 3.2 Anti‐OS effect of TB in vivo

Fluorescence intensity was used to assess the U2OS xenograft tumour growth in larval zebrafish. As shown in Figure 1A lower, a xenograft model of OS was successfully established in zebrafish and TB concentrations from low to high doses significantly inhibited the tumour growth with inhibitory rates from 24.6 to 27.3% as compared to untreated controls (all *P *<* *.05). Cis‐platinum also exerted significant inhibitory effect against the tumour growth (*P *<* *.05) at its NOAEL (75 μmol/L), which was no more effective than TB.

### 3.3 Inhibitory effect of TB in vitro

**Figure 2:**
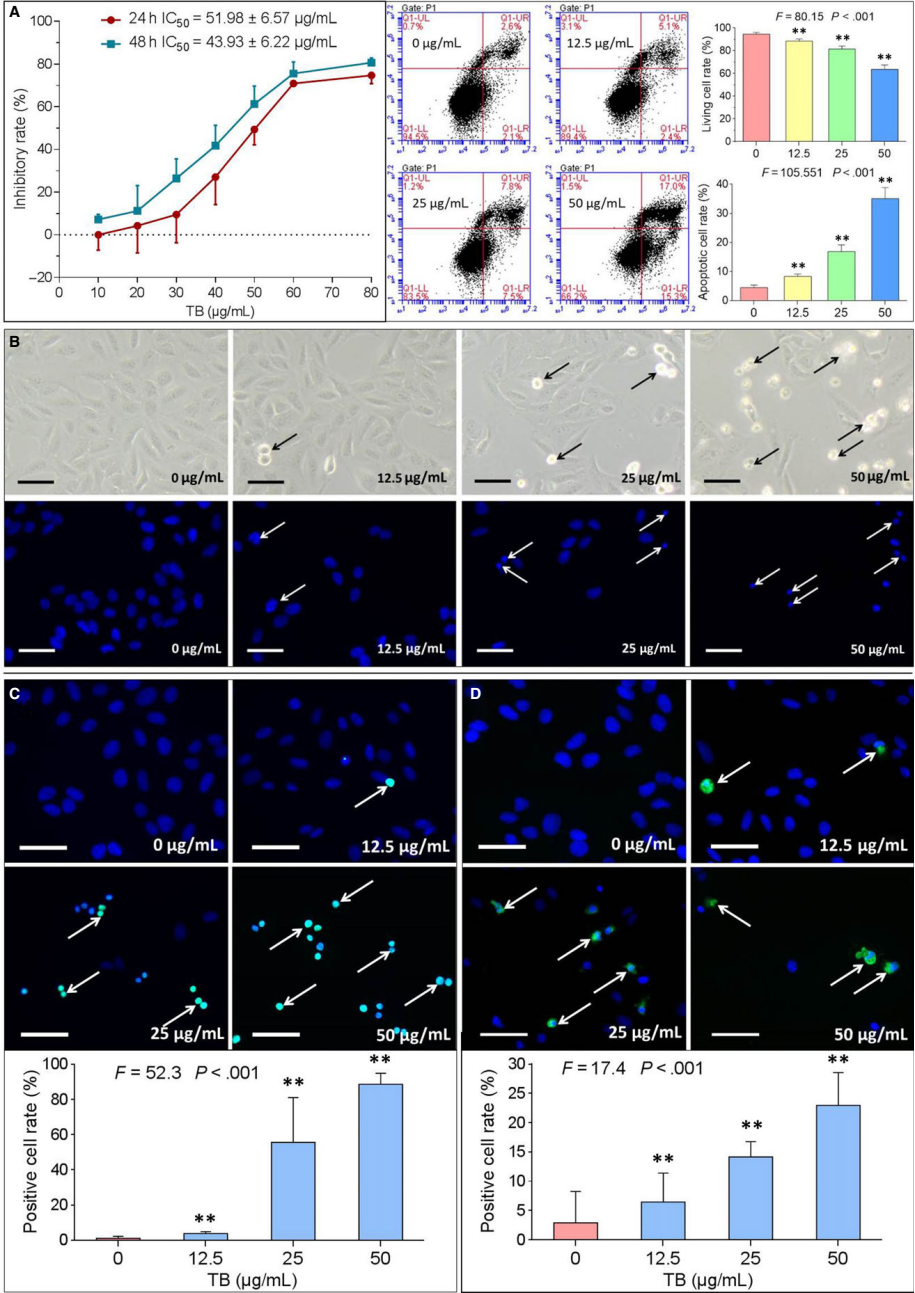
Effect of TB on U2OS cells. A, Cell viability (n* *=* *5) and flow cytometry analysis (n = 3). **P <* *.01 vs blank control. B, Apoptotic morphology by DAPI staining. Scale bar: 100 μm. C, Apoptosis assay by TUNEL staining. Scale bar: 100 μm. Values represent mean ± SD (n* *=* *5). **P <* *.01 vs blank control. D, Immunofluorescence assay of c‐Casp3 expression. Scale bar: 100 μm. Values represent mean ± SD (n* *=* *5). **P <* *.01 vs blank control

As shown by MTT assay in Figure 2A (left panel), TB induced an obvious inhibition on U2OS cell viability over its concentration range from 20 to 80 μg/mL at 24 hours and from 10 to 80 μg/mL at 48 hours. The inhibitory effect was more pronounced at increasing concentrations and increasing incubation times, indicating a concentration‐ and time‐dependent activity of TB. The IC_50_ values were 51.98 ± 6.57 μg/mL (24 hours) and 43.93 ± 6.22 μg/mL (48 hours). According to the IC_50_ values after 24 hours incubation, 12.5, 25 and 50 μg/mL were selected as low, intermediate and high doses of TB for the subsequent experiments.

### 3.4 Pro‐apoptotic effect of TB

After 24 hours treatment with TB, the morphology of U2OS cells was observed under a fluorescence microscope. As shown in Figure 2B, TB‐induced morphological changes typical for apoptosis (eg being round and shrunken). The number of abnormal cells increased with increasing concentrations. DAPI staining also revealed typical apoptotic signs (eg chromatin condensation, karyopyknosis and nuclear fragmentation) in U2OS cells upon TB treatment. Again, the number of apoptotic cells increased with increasing TB concentrations.

Flow cytometry using Annexin V‐FITC/PI staining was used to confirm the pro‐apoptotic effect of TB of U2OS cells. As shown in Figure 2A (right panel), increasing concentrations of TB for 24 hours significantly decreased the fraction of living cells from 94.6 ± 1.6% to 63.6 ± 3.8% (*P *<* *.001), while the fraction of apoptotic cells increased from 4.5 ± 0.9% to 35.1 ± 3.8% (*P *<* *.001).

By the TUNEL assay, we visualized and quantified in situ DNA strand breaks and fragments in nuclear chromatin of U2OS cells. This assay was performed to verify the pro‐apoptotic effects of TB. As shown in Figure 2C, apoptotic cells with positive fluorescein staining were detected in TB‐treated groups. The positive rate was significantly increased from 1.0 ± 1.2% to 88.7 ± 6.2% with increasing concentrations of TB, also indicating a concentration‐dependent reaction pattern.

To determine the specificity and safety of TB's pro‐apoptotic effects, normal larval zebrafish were treated and AO staining was performed. As shown in Figure 1C, TB induced no apoptosis in normal zebrafish larvae over its effective dose range (2.13 to 21.3 μg/mL) and even at a very high dose (MNLC, 213 μg/mL), whereas cis‐platinum exerted significant pro‐apoptotic effects in normal cells of zebrafish at its effective dose (NOAEL, 75 μmol/L). Hence, TB exerted pro‐apoptotic effects specifically to tumour cells but not to normal cells, indicating that TB was safer than chemotherapy with cis‐platinum.

### 3.5 Pro‐apoptotic targets of TB

**Figure 3:**
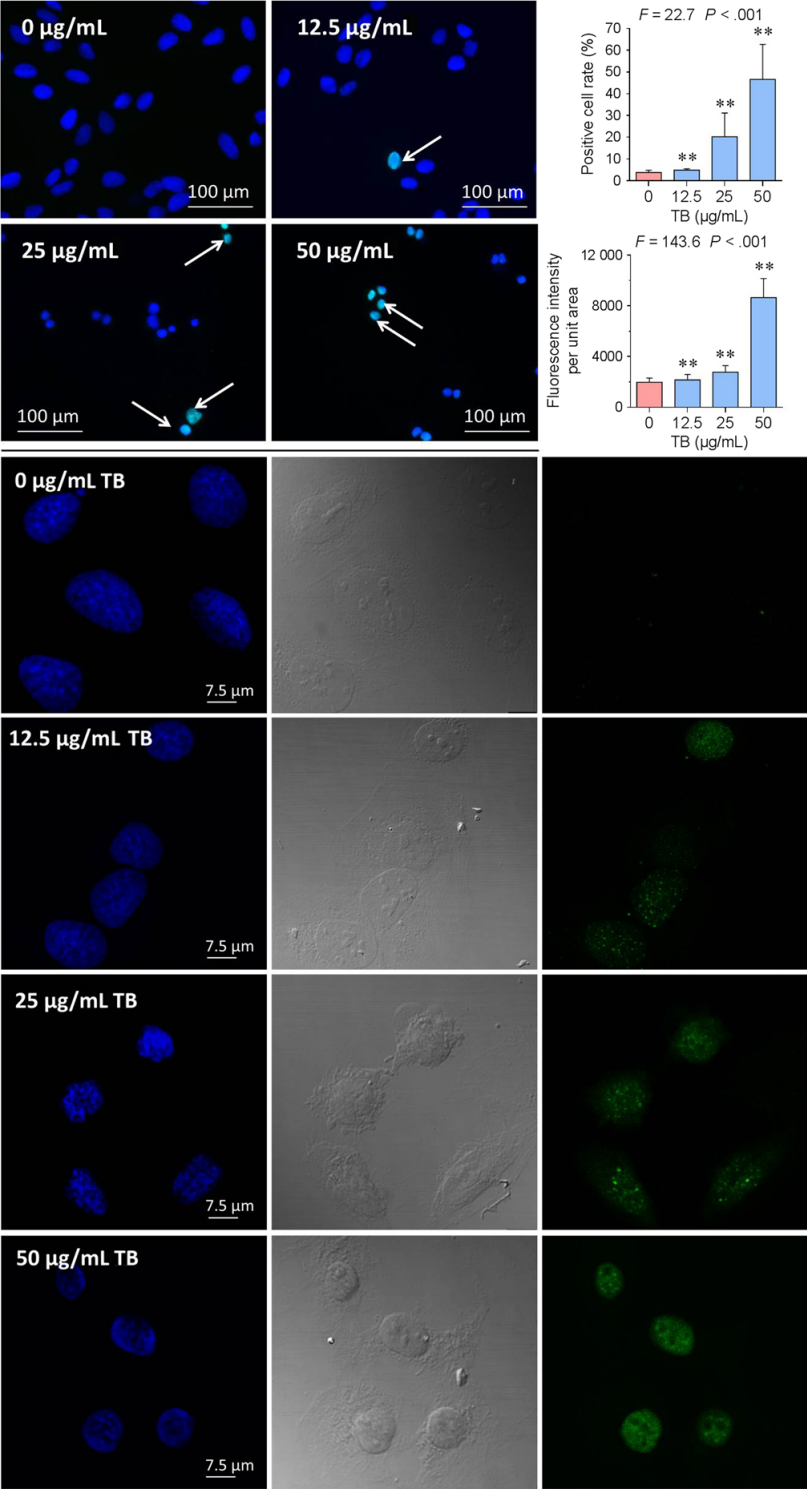
Detection of p‐H2AX expression in U2OS cells by immunofluorescence assay. Upper: Fluorescence microscopic observation. Lower: Confocal laser scanning microscopic observation. Values represent mean ± SD (n* *=* *5 and n* *=* *9). **P <* *.01 vs blank control

Immunofluorescence assay for cleaved‐caspase 3 (c‐Casp3) and phosphorylated H2AX (p‐H2AX) (Ser 139) was performed to study the pro‐apoptotic targets of TB on the cellular level. As shown in Figure 2D, the immunopositive rate of c‐Casp3 activated cells was significantly increased from 2.9 ± 5.4% to 22.9 ± 5.6% (all *P *<* *.01). As shown in Figure 3 upper, upon treatment with increasing concentrations of TB, p‐H2AX positive cells were detected by immunofluorescence. The fraction of immunopositive cells was significantly increased from 3.8 ± 1.0% to 46.6 ± 16.1% (all *P *<* *.01). The fluorescence of p‐H2AX positive cells was further observed and measured under a laser scanning confocal microscope, and the fluorescence intensity was found increased significantly with increasing concentrations of TB (Figure 3 lower). The above data indicated that TB‐induced apoptosis by activation of caspase cascade and triggered DNA damage (double‐strand breaks) through phosphorylation of H2AX in U2OS cells.

**Figure 4:**
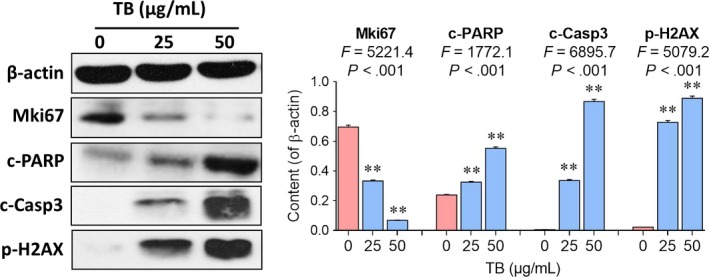
Western blot analysis of apoptosis‐related protein expressions in U2OS cells with TB treatment. Values represent mean ± SD (n* *=* *3). **P <* *.01 vs blank control

Western blot analyses using antibodies for Mki67, c‐PARP, c‐Casp3 and p‐H2AX were performed to determine the pro‐apoptotic targets of TB on molecular level. As shown in Figure 4, TB significantly inhibited the protein expression of Mki67 and increased the expression of c‐PARP, c‐Casp3 and p‐H2AX in U2OS cells in a concentration‐dependent manner (all *P *<* *.01).

### 3.6 Molecular mechanism of action of TB

**Figure 5:**
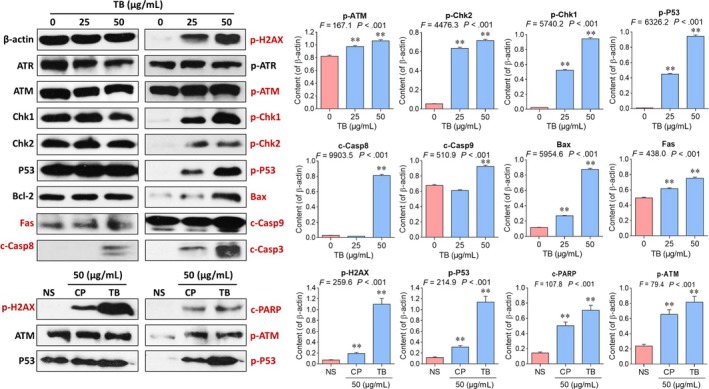
Western blot analysis of p53 signalling‐related protein expressions in U2OS cells. NS: untreated group; CP: cis‐platinum group. Values represent mean ± SD (n* *=* *3). **P <* *.01 vs blank control

Since c‐Casp3 and p‐H2AX were activated by TB, the p53 signalling pathway could be deduced as a main regulator of TB's anti‐OS effect. To verify this assumption, Western blot assays were performed using antibodies against upstream and downstream signalling components of the p53 signalling pathway. As shown in Figure 5, TB treatment from 0 to 50 μg/mL significantly increased the phosphorylation levels of p53 and its upstream molecules ATM, Chk1, Chk2 (all *P *<* *.01). The expression of the downstream proteins Bax, Fas, c‐Casp9 and c‐Casp8 of P53 were also increased by TB. Moreover, as compared with cis‐platinum, TB activated more expressions of p‐H2AX, c‐PARP, p‐ATM and p‐P53, indicating that TB has stronger effect than that of the “classical” DNA damaging agent.

**Figure 6:**
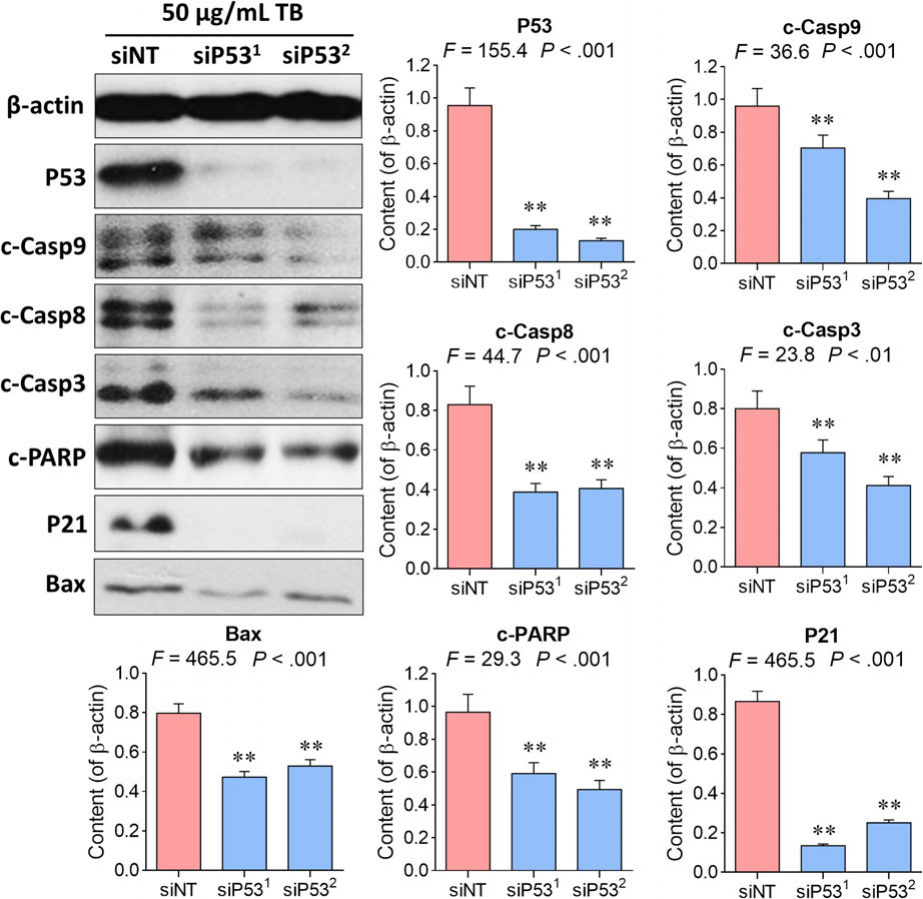
Western blot analysis of protein expressions in U2OS cells with TB (50 μg/mL) and *TP53‐siRNA* treatment. Values represent mean ± SD (n* *=* *3). **P <* *.01 vs blank control

*TP53‐siRNA* was used to verify the regulatory role of the p53 signalling pathway in TB‐induced apoptosis. As shown in Figure 6, with high dose of TB (50 μg/mL) and *TP53‐siRNA* treatment, p53 was not detectable anymore and the expressions of its downstream molecules c‐Casp9, c‐Casp8, c‐Casp3, P21 and Bax were significantly decreased (all *P *<* *.01). Furthermore, c‐PARP, the marker of caspase‐3 mediated apoptosis, was also inhibited (*P *<* *.01), indicating an inhibition of apoptosis by *TP53‐siRNA*.

### 3.7 Effect of TB on p53‐mut OS cells and normal cells

**Figure 7:**
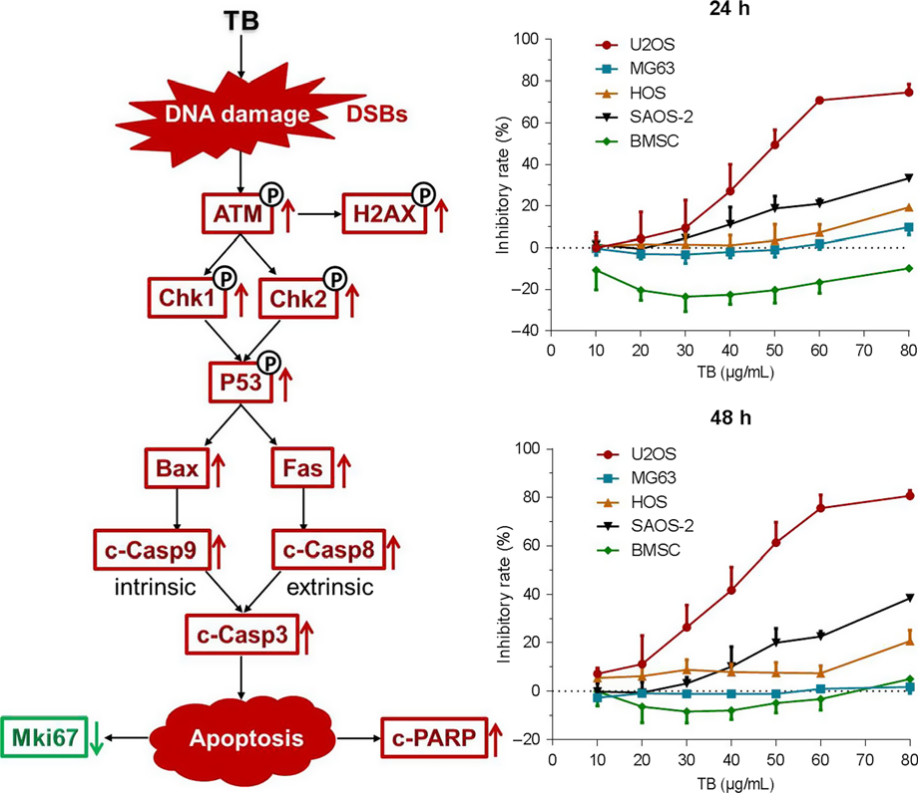
Left: TB‐activated p53 signalling pathway in U2OS cells. Right: Cell viability of various cell lines with TB treatment (n* *=* *5)

As shown in Figure 7 (right panel), TB significantly inhibited the viability of SAOS‐2 cells from 50 to 80 μg/mL at 24 hours and 48 hours, indicating a concentration‐dependent manner over its effective dose range. However, TB showed little effect on MG63 and HOS cells over its concentration range from 10 to 60 μg/mL, and only a slight inhibitory tendency was seen with TB at 80 μg/mL. In contrary, TB was found to increase the viability of BMSC from 10 to 80 μg/mL at 24 hours and from 20 to 60 μg/mL at 48 hours, indicating a beneficial effect on the normal cells.

## 4 DISCUSSION

Natural products have attracted growing interest in anticancer therapy due to their advantages of high bioactivity and low toxicity.^35^, ^36^ As one of the most commonly used natural products, tea possesses therapeutic potential for treating cancers, including OS.^30^, ^37^, ^38^ The components, such as polyphenols, polysaccharide, pigments (TB, TF, and TR), etc., may be responsible for the anticancer activity of tea.^39^ To date, a large number of studies have focused on tea polyphenols (mainly catechins) and revealed their chemopreventive and adjuvant effects on cancer.^40^, ^41^ However, poor bioavailability, unfavourable side effects, and high costs limited the development and clinical application of catechins.^42^, ^43^, ^44^ Apart from polyphenols, TB attracts increasing attention in recent years due to its bioactivities and therapeutic potential.^32^, ^45^ In our previous studies, TB exerted strong pro‐apoptotic effects against non‐small cell lung cancer via the p53 signalling pathway.^33^, ^34^ Using xenograft model and osteosarcoma cells, this study investigated the anti‐OS effects and molecular mechanism of TB.

The in vivo data showed that TB exerted a significant anti‐OS effect at doses ranging from 1/10 NOAEL to NOAEL levels. The effect was stronger than that of cis‐platinum at its NOAEL level. The in vitro data revealed that TB‐inhibited proliferation and induced apoptosis of U2OS cells in a concentration‐dependent manner. Through immunofluorescence and Western blot assays, we determined the protein effectors of TB as Mki67, PARP, Casp3 and H2AX. Mki67 is a 359 kD nuclear protein for ribosomal RNA transcription, which can be used as a marker of cell growth and proliferation.^46^, ^47^ Inactivation of Mki67 leads to suppression of ribosomal RNA synthesis and thereby induces inhibition of cell proliferation,^48^ just as what TB did in this study. PARP is a 116 kD nuclear poly (ADP‐ribose) polymerase responsible for DNA repair and cell viability in response to exogenous stress.^49^ It mainly consists of 4 domains: DNA‐binding domain, caspase‐cleaved domain, auto‐modification domain and catalytic domain. The catalytic domain can be efficiently cleaved from DNA‐binding domain by interleukin‐1 converting enzyme (ICE)‐like caspases (mainly caspase 3), resulting in cleavage of PARP.^50^, ^51^ The cleavage facilitates cellular disassembly and serves as a marker of cell apoptosis, and the amount of cleaved PARP (c‐PARP) can be used to assess the propensity of cells to apoptosis.^52^ Caspase 3 is one of the key executioners of apoptosis, which is either partially or totally responsible for the proteolytic cleavage of PARP.^53^ Activation of caspase 3 requires proteolytic processing of its inactive zymogen into activated p17 and p21 fragments, and the amount of cleaved caspase 3 (c‐Casp3) is a quantitative index of apoptosis induction.^50^ Our data showed increased expression of both c‐PARP and c‐Casp3, indicating a caspase3‐activated PARP cleavage in response to TB‐induced apoptosis. The histone H2AX, a member of the H2A protein family, is a subunit of the histone octomer in nucleosomes. If endogenous or exogenous DNA damage causes double stranded breaks (DSBs), H2AX will be phosphorylated by kinases such as ATM (ataxia telangiectasia mutated) and ATR (ATM‐Rad3‐related).^54^ p‐H2AX is thought to be the first step in recruiting and localizing DNA repair proteins as the first response to DSBs. Therefore, p‐H2AX can serve as a biomarker for early DNA damage.^54^, ^55^ Our data showed p‐H2AX formation upon TB treatment but none in untreated control, indicating DNA damage related to DSBs in U2OS cells. Our data indicated that TB initially triggered DNA damage with phosphorylation of H2AX and subsequent activation of caspase 3, followed by cleavage of PARP and inhibition of Mki67, resulting in cell proliferation inhibition and apoptosis induction in U2OS cells. Moreover, the phosphorylation of H2AX gave a clue that either ATM or ATR might be involved in the DNA damage response to TB. Since p53 is the main substrate of ATM or ATR and is responsible for cell apoptosis, the p53 signalling pathway could be assumed mediating the pro‐apoptotic mechanism of TB.

To verify this assumption and clarify TB's underlying mechanism, we analyzed the molecules on p53 pathway related and confirmed an activation of p53 signalling pathway in TB‐treated U2OS cells. ATM is a 350 kD serine/threonine kinase that regulates DNA repair.^56^ Activation of ATM by autophosphorylation on Ser1981 occurs in response to exposed DNA DSBs, and the activated ATM (p‐ATM) activates p53 by phosphorylating downstream Chk1 and Chk2 for apoptosis induction.^56^, ^57^ Chk1 is a serine/threonine‐specific protein kinase that plays an important role in DNA damage checkpoint control and tumour suppression.^58^ Activation of Chk1 involves phosphorylation at Ser317 and Ser345 by ATM and occurs in response to blocked DNA replication and certain forms of genotoxic stress.^59^ Chk2 is the mammalian orthologue of the budding yeast Rad53 and fission yeast Cds1 checkpoint kinases. It induces apoptosis as a tumour suppressor in an ATM‐dependent manner.^60^, ^61^ The amino‐terminal domain of Chk2 contains a series of serine or threonine residues which are known to be preferred sites for phosphorylation by ATM kinase.^62^ Following DNA damage, p53 can be phosphorylated by Chk1 and Chk2 at Ser15 and Ser20 through the ATM kinase cascade.^63^ Phosphorylated p53 (p‐P53) induces apoptosis through activating transcription of hundreds of molecules, including the members of Bcl‐2 family, TNFR family and caspase family.^64^ In this study, the downstream Bax, Fas, Casp8, Casp9 and Casp3 were activated with increased levels of p‐P53. Therein, Bax is a pro‐apoptotic Bcl‐2 family member that resides in the cytosol and translocates to mitochondrial membrane after activation, acting as a key component for cellular‐induced apoptosis through mitochondrial stress.^65^, ^66^ Upon apoptotic stimulation, Bax interacts with pore proteins on the mitochondrial membrane to increase the membrane's permeability, leading to the release of cytochrome *c* from mitochondria and activation of caspase 9 (Casp9).^67^, ^68^ Fas, as another transcriptional target of p53, belongs to the TNFR family and triggers the formation of a death‐inducing signalling complex (DISC) involving the recruitment of caspase 8 (Casp8).^69^ Activation of Casp8 and Casp9 initiates a caspase cascade to activate caspase 3 (Casp3), leading to both intrinsic (mitochondrial) and extrinsic caspase‐dependent apoptosis of U2OS cells.

P53 is a DNA‐binding tumour suppressor which serves as a cornerstone in the cellular response to stress and in particular in the defence against malignant transformation.^64^, ^70^ It activates apoptosis‐related genes to mediate programmed cell death, cell cycle control and cell apoptosis mechanisms. If the DNA damage is limited, the activation of p53 induces a cellular programme to allow DNA repair, whereas in case of severe DNA damage, the p53 response will drive the cell into apoptosis.^70^ TB can thereby be regarded to induce a severe DNA damage in U2OS cells by activation of p53 signalling pathway. When p53 was knockdown by *TP53*‐siRNA, the signalling was blocked and the apoptosis suppressed due to the inhibition of its downstream caspases (c‐Casp9, c‐Casp8 and c‐Casp3) and c‐PARP. This result verified the key role of p53 in TB‐induced apoptosis in U2OS cells and indicates a p53‐dependent mechanism. It is well known that p53 mutations are involved in many cases of human osteosarcomas and other cancers. Thus, we applied p53 mutant cell lines (HOS, SAOS‐2 and MG63) to determine whether or not the TB′s anti‐OS effect was p53‐dependent. We found that TB has stronger inhibitory effect on the p53‐wt U2OS cells than that on the p53‐mut cells, but it could also inhibit SAOS‐2 cells (p53 null) obviously at its high concentrations. The result indicated a p53‐dependent mechanism on the p53‐wt cells and a p53‐independent mechanism on the p53‐mut cells. Further studies are needed to clarify the latter mechanism of TB.

During recent years, zebrafish xenograft models have been increasingly generated to study malignancies, qualifying this illustrative animal system for the study of human cancers.^71^ Some experiment‐specific characteristics of zebrafish make it superior to other model systems. The comparative advantages are as follows: (i) the zebrafish genome has been fully sequenced, showing many conserved genes as compared to the human genome^72^; (ii) No immune rejection against human cells in larval zebrafish supports the xenograft model by xenotransplantation^73^; (iii) The transparency of larval zebrafish provides an advantage of real time visible observation^74^ (iv) Large‐scale generation and rapid organogenesis provides a short experimental period and a high‐throughput manner^75^; and (v) Zebrafish are vertebrate animals with developmental processes comparable to the human development and have remarkable physiological and pharmacological homologies with human beings.^76^ More importantly, zebrafish are specifically suitable for OS models and anti‐OS studies, because OS commonly occurs in children and young adults which can be better mimicked by zebrafish at the larval stage. Therefore, we applied larval zebrafish to establish xenograft OS model. More importantly, zebrafish are very sensitive to toxic stimuli, which can be used for prediction of potential side effects of novel drugs. Our data revealed that TB at 1/10 NOAEL level exerted comparable pro‐apoptotic effect (24.6%) than that of cis‐platinum at NOAEL level (24%). Besides, TB at MNLC level exerted much lower side effects on normal tissues/cells than that of cis‐platinum at NOAEL level. This is a strong clue for the advantage of TB over chemotherapy. The effective doses (from 2.13 to 21.3 μg/mL) in zebrafish can be estimated as 0.1 to 1.0 mg/kg in human by dose conversion,^77^ which are very low and applicable for clinical application. Further, we applied BMSC to verify the safety of TB on normal cells and found that TB not only exerted no cytotoxicity on BMSC, but also increase the viability of BMSC. Since BMSC is a p53‐wt cell line as U2OS, a question has been raised as to why TB‐induced DNA damage and apoptosis through p53 signalling pathway only in U2OS cells rather than the p53‐wt normal cells. The answer lies in the fact that the DNA damage response functions differently in cancer cells than it does in normal cells.^78^ Unlike normal cells, cancer cells maintain a marked level of genomic instability and will be associated with replication stress factors such as cell cycle checkpoint loss, increased transcription, higher levels of metabolic stress, increased ROS formation and activation of oncogenic drivers.^78^ The principle hallmarks of cancers are their propensity to accumulate DNA damage and their decreased traditional repair capacity, leading cancer cells to become exceedingly more dependent on homologous recombination repair as a means of protection from the lethal effect of both spontaneous and therapy‐induced DSBs in DNA.^79^ Therefore, DNA‐damaging regimes, such as TB, could selectively induce tumour cell apoptosis by augmenting genomic instability, and the DNA repair mechanisms would work efficiently in normal cells in contrast to tumour cells.

Although TB exerted better anti‐OS efficacy outcomes than cis‐platinum, the OS tumour still existed in zebrafish after the treatment. It reminds us to consider the combination of TB and chemotherapy for enhancing the efficacy and reducing the toxicity in future studies. Taken together, our results indicated that TB‐exerted anti‐OS effects as a pro‐apoptotic agent through p53 signalling pathway. It can be regarded as an important target for TB on p53‐wt cancers.

## CONFLICTS OF INTEREST

There is no conflict of interest for this work.

## AUTHOR CONTRIBUTIONS

Wangdong Jin and Li Zhou performed the main experiments of this study; Bo Yan, Li Yan, Fucun Liu, Wenhua Yu and Min Lv contributed to the materials acquisition and data analysis for this study; Yiqiao Xu and Chunqi Li contributed to the zebrafish‐related experiment for this study; Li Xie contributed operated the confocal laser scanning microscope; Letian Shan designed the main part of this work and drafted the manuscript; Peijian Tong and Qiang Yuan improved the design and provided funding support to this work; Thomas Efferth improved the design, draft and revision of this work. All listed authors approved the manuscript for publication, and agreed to be accountable for all aspects of this work.
